# Supplementary material for: Proximity Effect of Optically Active h-BCN Nanoflakes Deposited on Different Substrates to Tailor Electronic, Spintronic, and Optoelectronic Properties
Source: Int J Mol Sci. 2025 Feb 27;26(5):2096. doi: 10.3390/ijms26052096 (PMC11899974; doi:10.3390/ijms26052096)
Supplement: Supplementary file 1 [file ijms-26-02096-s001.zip › ijms-3444550-supplementary.pdf]

Supplementary materials

Symmetry effect on Exchange interaction in 6-atom decorated model

The simple toy model of the interaction between the chiral h-BCN and magnetic adatoms can be constructed by placing a single adatom on top of a tri-fold B-N structural unit as shown in Figure S1. This configuration forms a hexamer of atoms with local spin moments. Due to lack of the inversion symmetry in this magnetic systems, we anticipate that DMI interactions alongside the spin-orbit (SO) and Rashba spin-orbit coupling will modify the magnetic texture of hexamer. These interactions may give rise to chiral magnetism, providing an opportunity to investigate potential coupling between the chiral structure of the non-magnetic nanoflake and the chiral magnetic properties of the TM layer.

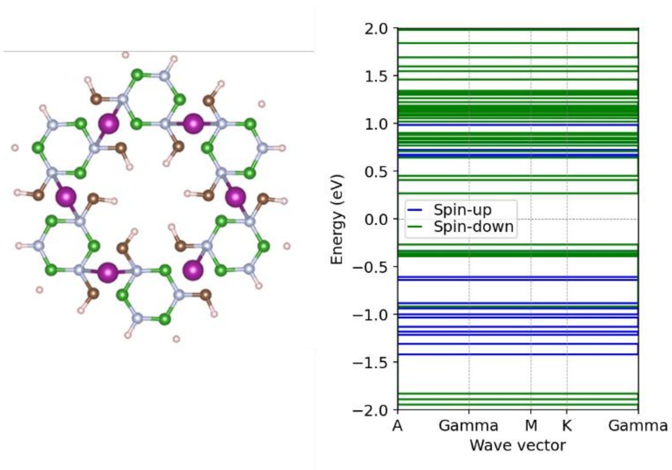

Figure S1. MO diagram of the Mn decorated h-BCN nanoflake.

The magnetic Hamiltonian can be written as follows:

$$H = -\sum_{<ij>} J_{ij}\, \vec{\boldsymbol{S}}_{\boldsymbol{i}} \cdot \vec{\boldsymbol{S}}_{\boldsymbol{j}} + \sum_{<ij>} \overleftrightarrow{\boldsymbol{D}}_{\boldsymbol{ij}} \cdot (\vec{\boldsymbol{S}}_{\boldsymbol{i}} \times \vec{\boldsymbol{S}}_{\boldsymbol{j}}) \ldots\ldots\ldots\ldots\ldots\ldots\ldots\ldots\ldots\ldots S_1$$

TB2J Conventions of Heisenberg Model

$$H = -\sum_i K_i \overrightarrow{\boldsymbol{S}_{\boldsymbol{i}}^{\boldsymbol{2}}} - \sum_{i\neq j} [J_{ij}^{iso}\ \vec{\boldsymbol{S}_{\boldsymbol{i}}} \cdot \vec{\boldsymbol{S}_{\boldsymbol{j}}} + \vec{\boldsymbol{S}_{\boldsymbol{i}}} J_{ij}^{ani} \vec{\boldsymbol{S}_{\boldsymbol{j}}} + \vec{D_{ij}} \,.\, \left(\vec{\boldsymbol{S}_{\boldsymbol{i}}} \times \vec{\boldsymbol{S}_{\boldsymbol{j}}}\right) \ldots\ldots\ldots S_2$$

where $\vec{\boldsymbol{S}}_{\boldsymbol{i}}$ is the unit vector of the magnetic moment at site i, $\vec{\boldsymbol{S}}_{\boldsymbol{j}}$ is the unit vector of the magnetic moment at site j, J_{ij} is the Heisenberg exchange, $\overleftrightarrow{\boldsymbol{D}}_{\boldsymbol{ij}}$ is the antisymmetric Dzyaloshinskii-Moriya interaction parameters, while K_i is uniaxial anisotropy parameter. We are using notations in convention with TB2J code. The exchange parameter J_{ij} are calculated using perturbation theory. J_{ij} between spins at sites i and j is often computed using the following expression:

$$J_{ij} = \frac{1}{4}\sum_{mn}\left(\frac{\partial^2 E}{\partial\theta_i\,\partial\theta_j}\right)_{mn} \ldots\ldots\ldots\ldots\ldots\ldots\ldots\ldots\ldots\ldots\ldots\ldots\ldots\ldots\ldots\ldots\ldots\ldots\ldots.. \ldots\ldots . S_3.$$

where H is the total energy, and θ_i and θ_j are the angles of the spins at sites i and j. We find a strong dependence of the exchange interactions depending on the nature of the adsorbing atoms as well as the substrate.

Symmetry makes significant changes in the details of exchange interactions. This particularly concerns the DMI interaction. Because DMI interaction vector has specific direction that is strongly affected by the symmetry of the interface. Particularly, the planar hexamer of TM sites has DMI vectors in the xy-plane of the hexamer directed radially towards/away from its center. However, when TM hexamer located at the top of the chiral h-BCN, DMI vectors are now have large z-component, and xy-components are strongly affected by the distortions appearing due to the interactions of TM with the substrate. The strong modifications of the DMI leads to non-collinear magnetic textures. The considered system is somewhat small and we find that exchange coupling is stronger than the DMI. We observe a slight variation in the direction of magnetic moments from collinear arrangement.

Table S1. The exchange parameters for Ni hexamer.

Pair ij	r_{ij}	J_{ij}, (meV)	$\lvert\overleftrightarrow{\boldsymbol{D}}_{\boldsymbol{ij}}\rvert$ (eV)	DMI $\vec{e}$ (x,y,z)*
Ni 2-1	4.631	-11.10	0.8235	(0.79, 0.23, 0.00)
Ni 3-2	4.335	-8.95	0.95	(0.61, -0.73, 0.03)
Ni 4-3	4.16	-8.40	1.34	(-0.32, -1.30, -0.02)
Ni 6-1	4.46	-11.71	1.19	(-0.21, -1.17 , 0.07)
Ni 6-5	4.48	-11.70	0.96	(-0.60, 0.72, -0.02)
Ni 5-4	4.50	-11.49	0.88	(-0.83, -0.29, -0.08)
Ni 4-3	4.16	-8.4	1.36	(-0.32, -1.31, -0.02)

* $\vec{e}$ (x,y,z) is direction vector for DMI parameters.

The exchange parameters for Co hexamer.

Pair ij	r_{ij}	J_{ij},((meV)	$\lvert\overleftrightarrow{\boldsymbol{D}}_{\boldsymbol{ij}}\rvert$ (eV)	DMI $\vec{e}$ (x,y,z)
Co 4-3	4.625	37.46	15.63	(-9.64, -7.97, -9.37)
Co 6-5	4.760	26.36	26.08	(-15.24, -12.26,-17.24)
Co 3-2	4.760	15.28	30.73	(-15.91, -16.62, -20.36)
Co 2-1	4.761	13.18	30.92	(-17.58, -16.14, -19.64)
Co 6-1	4.812	20.84	30.32	(17.36, 14.033, 20.526)
Co 5-4	4.902	12.59	34.29	(-19.87, -16.23, -22.74)

Anisotropy changes between Co and Ni hexamer

Finally, depending on the TM the anisotropy of the system governs the magnetic moment of antisymmetric system. Specifically, we observe in-plane magnetization for Co, while out of plane magnetization for Ni. The magnetocrystalline anisotropy energy (MAE) is -0.008 and +0.003eV, respectively.
